# Pseudo-Pulseless Electrical Activity With Anaphylaxis in a Pediatric Surgical Patient: A Case Report

**DOI:** 10.7759/cureus.112837

**Published:** 2026-07-17

**Authors:** Joshua Stopak, Martha O Herbst, Alexandria R Nicolaus, Timothy W Morris

**Affiliations:** 1 Department of Anesthesia, University of Iowa, Iowa City, USA

**Keywords:** allergy and anaphylaxis, anaphylactoid reaction, pediatric anesthesia, pediatric resuscitation, pseudo-pulseless electrical activity

## Abstract

Pseudo-pulseless electrical activity (p-PEA) is a clinical condition where cardiac output drops severely enough to produce a non-palpable pulse in the setting of organized electrical activity of the heart, but imaging or monitors display evidence of cardiac motion. It may be seen in shock states such as anaphylactic shock.

We describe a case where a previously healthy teenage patient presenting for operative removal of orthopedic hardware developed p-PEA after anaphylaxis to antibiotics. Despite loss of pulses and severe mottling of the skin, the patient’s pulse oximetry (plethysmography) and capnometry (measurement of end-tidal carbon dioxide) displayed visually normal, reassuring waveforms. The condition responded to chest compressions and administration of epinephrine and vasopressin; the patient was ultimately able to be discharged home on postoperative day (POD) 2.

Prompt recognition, diagnosis, and clinical management of p-PEA and anaphylaxis are key to good patient outcomes. Epinephrine is the mainstay of treatment for anaphylactic reactions and is indicated during cardiopulmonary resuscitation (CPR) for p-PEA.

## Introduction

Pseudo-pulseless electrical activity (p-PEA), when compared to classical pulseless electrical activity (PEA), or the absence of a palpable pulse in the presence of organized electrical activity on ECG monitoring, is understood as a state of reduced cardiac output in which a palpable pulse is not present, but some cardiac motion is still present [1]. Its clinical differences from PEA have been noted with the greater use of point-of-care ultrasound (POCUS) during resuscitations, which can reveal the presence or absence of cardiac wall motion. While there remains limited evidence regarding the prevalence and survivability of p-PEA, it has been reported to potentially represent up to 41% of cases of presumed PEA, which is theorized to be the reason for the higher survivability of PEA versus asystole [1,2]. Pseudo-PEA is also defined as the presence of an aortic pulse in the setting of a perfusion pressure of less than 40 mm Hg, which can be seen in severe hypovolemia, pulmonary embolism, tension pneumothorax, cardiac tamponade, septic shock, or neurogenic shock [3]. 

## Case presentation

A 16-year-old female patient weighing 62 kg with a history of astigmatism, leg length discrepancy, and femoral anteversion of the right lower extremity presented for elective hardware removal. Prior surgical history included epiphysiodesis of the distal femur and one revision (two prior surgeries at our institution). She had no history of medication allergies. She received an American Society of Anesthesiologists classification score of 1. After intravenous (IV) induction of anesthesia with 2 mg of midazolam, 50 mcg of fentanyl, 40 mg of lidocaine, and 200 mg of propofol, a size 4 intubation-compatible supraglottic airway was placed and anesthesia was maintained with sevoflurane. Muscle relaxant medications were not administered during induction. 

A few minutes after induction, the patient received 6 mg of dexamethasone and 1,750 mg of cefazolin intravenously. During surgical preparation, about 25 minutes after administration of induction medications and 22 minutes after administration of the antibiotic and steroid, the surgeon noted mottling of the lower extremity, which progressed to generalized mottling of the whole body. Concomitant hypotension was noted and treated initially with phenylephrine (50 mcg 16 minutes after induction medications, then 100 mcg three minutes later, then 200 mcg two minutes later, then 300 mcg four minutes after the previous dose; a phenylephrine infusion with rates ranging from 0.5 to 1 mcg/kg/min was initiated 20 minutes after induction medications were given). Blood pressure continued to decrease to a nadir of 50/26, and then the cuff cycled without displaying a numeric value for several minutes. Heart rate progressively increased throughout the episode, trending from 60 to 120. Epinephrine 5 mcg was also given 28 minutes after induction, after peripheral pulses were nonpalpable despite reassuring end-tidal capnometry and pulse oximetry waveforms. No urticaria, angioedema, wheezing, or difficulty ventilating was noted. Potentiating agents for hypotension, including sevoflurane gas, were stopped, and the patient was ventilated with 100% oxygen.

**Figure 1:**
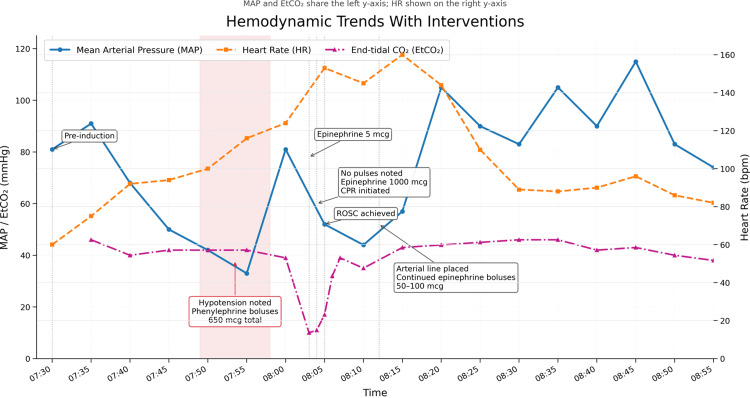
Patient monitoring data and interventions during the case. ROSC: return of spontaneous circulation; CPR: cardiopulmonary resuscitation

One two-minute round of cardiopulmonary resuscitation (CPR) was given 30 minutes after induction due to no palpable central pulses, and return of spontaneous circulation (ROSC) was achieved (Figure 1). Epinephrine 1,000 mcg and rocuronium 100 mg IV were administered at this time, and the supraglottic airway was exchanged for a cuffed single-lumen oral endotracheal tube due to inefficient ventilation through the supraglottic airway during chest compressions. End-tidal carbon dioxide was relatively stable around 40 mm Hg throughout the episode, other than the reduced readings observed during CPR prior to placement of the endotracheal tube. An arterial line and a central line were placed. Serum tryptase obtained at one hour (8.7 ng/mL) and two and a half hours (7.0 ng/mL) after the event was within normal limits (institutional laboratory reference range, <11.5 ng/mL). Hemodynamics improved, and the patient was able to be titrated off pressors and extubated later that same afternoon, about four hours after the initial event. Allergy and immunology consultants suspected hypersensitivity to one or more intraoperative medications. Given the timing of administration, propofol and rocuronium were thought to be less likely causative agents. Review of prior anesthetic records indicated the patient had previously received all administered medications, including cefazolin (two prior exposures) and rocuronium (one prior exposure).

**Figure 2:**
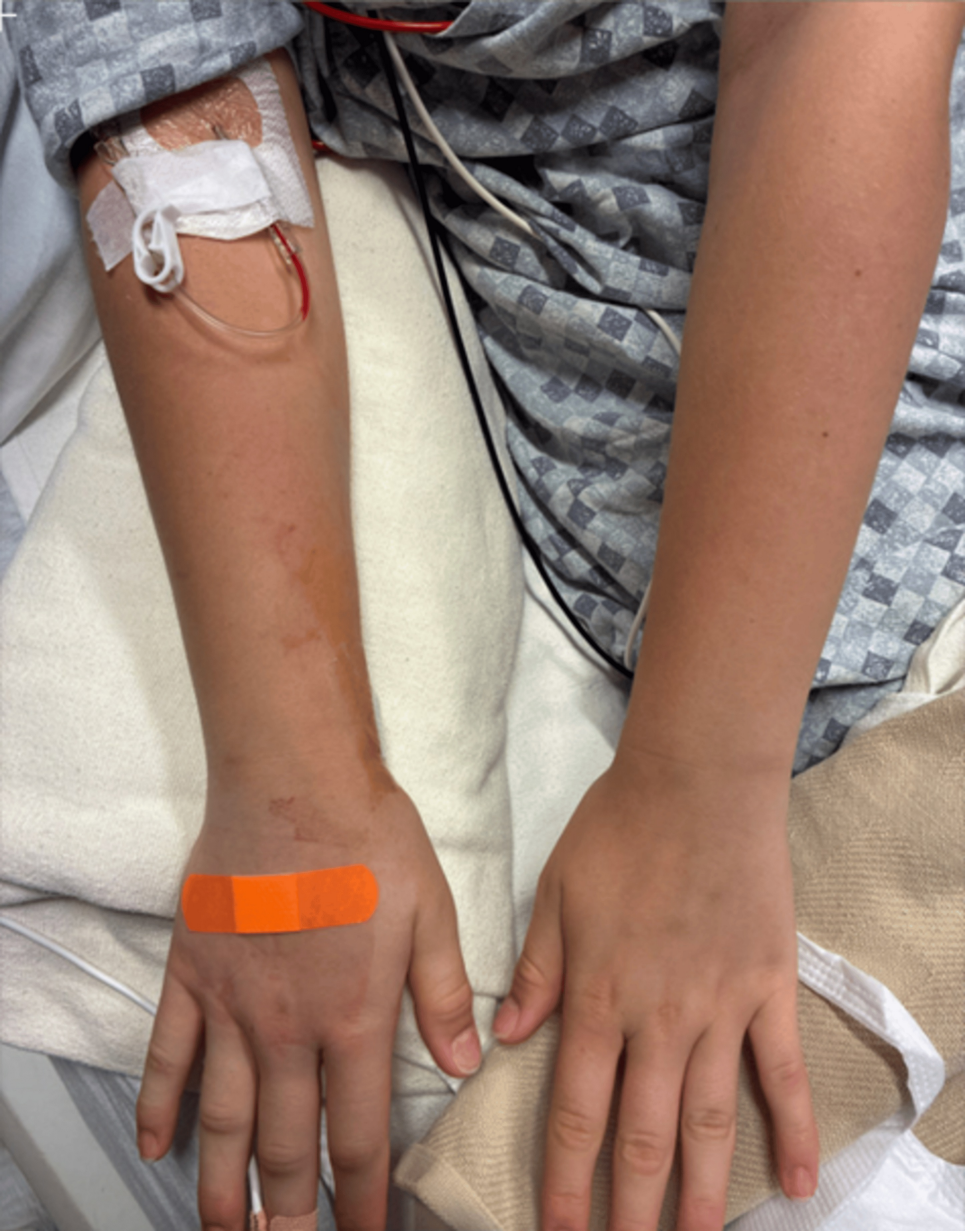
Right hand and arm swelling.

Examination of the patient on postoperative day (POD) 1 revealed that the patient reported a sore throat that was amenable to acetaminophen and ibuprofen administration and to sipping cold drinks. She continued to deny chest pain or discomfort but did note increased swelling in the right arm (Figure 2). The arm was warm, well perfused, with minor erythema, and was noted to be one centimeter larger in diameter than the contralateral arm. A vascular duplex examination was performed, revealing a focal segment of acute superficial thrombus in the right cephalic vein around the tip of the IV at the antecubital fossa. Hematology was consulted and recommended low-dose apixaban 2.5 mg twice daily. The right arm IV was also removed. The patient tolerated treatment well and was discharged the morning of POD 2.

On her outpatient follow-up visit three months after the case, the patient's limbs were noted to be normal. Her cefazolin skin testing came back positive, with a 7 mm wheal and 30 mm flare (saline control, 0 mm); all other suspected agents, including lidocaine, propofol, rocuronium, dexamethasone, midazolam, and fentanyl, were negative. At this outpatient visit, her baseline tryptase was 1.8 ng/mL. Based on the formula ($(\mathrm{baseline} \times 1.2) + 2$) [4], anything over 4.16 would indicate mast cell activation. Given that her tryptase levels were 8.7 and 7 during the event and, in light of her hemodynamic symptoms and positive skin testing, this is consistent with perioperative anaphylaxis. When this patient returns for her hardware removal surgery, avoidance of cefazolin in favor of an alternate antibiotic, such as vancomycin or clindamycin, is advised [5].

## Discussion

Much of the recent literature regarding p-PEA is focused on the adult population. Limited literature discusses the identification and management of p-PEA in infants in the NICU, and there remains a paucity of literature related to its identification and management during anesthesia [1]. In adult cardiac arrest, about 20% of arrests are due to PEA. In an analysis of adult PEA arrests, 18% were found to be p-PEA, and those with p-PEA, compared to those with true PEA, had a 4.4-fold higher chance of achieving ROSC (RR 4.35, 95% CI 2.20-8.63, p < 0.00001) [6]. The rate of PEA in newborns and infants has been reported to be 1%-5%; however, the incidence of p-PEA remains unknown [1]. This lack of published recognition of p-PEA in the pediatric population, along with the limitations of Pediatric Advanced Life Support (PALS) and the Neonatal Resuscitation Program (NRP), which rely solely on ECG, has, in part, led to increasing recommendations for POCUS inclusion in resuscitative protocols [1,7,8]. Delayed epinephrine administration is associated with non-survival, while porcine models show improved outcomes in p-PEA when epinephrine is combined with CPR [9]. Utilization of POCUS in resuscitative protocols would allow for earlier identification and treatment of p-PEA.

In modern anesthesia practice, there is greater availability of increasingly advanced monitoring and displays. We recommend using monitor data as one component of the information when assessing complete patient status; synthesizing monitor output while prioritizing findings from real-time physical examinations can optimally guide patient care. Anesthesia has been performed on patients for centuries, with new advances in technology and information. Throughout this time, anesthesiologists have used clinical judgment based on multiple factors in the operating room and the patient's condition, rather than singular monitor signs [10]. Synthesizing real-time patient status based on clinical judgment and deciding which monitor readings should be given critical consideration and which can be considered "artifact" in the setting of otherwise reassuring data and observations is a skill set that comes with time and practice in anesthesia [11]. We agree with Buhre and Rossaint's conclusion that the "presence of an appropriately trained and experienced anesthetist is the main determinant of patient safety during anesthesia" [12].

Peripheral administration of pressors may be less noxious than traditionally taught, provided good IV line stewardship is applied in placing and maintaining the line [13]. For pressor use, a hand IV is less preferred than an IV placed more proximally on the extremity. A larger-gauge IV line may be preferable, as well as placement under ultrasound guidance [14]. Our patient had a 20-gauge IV catheter in the hand, which showed signs of swelling after pressor (phenylephrine, epinephrine, vasopressin) administration but ultimately resolved without signs of necrosis.

In patients with suspected anaphylaxis, serum tryptase is the most widely utilized biomarker to support the diagnosis of anaphylaxis, reflecting mast cell activation and typically peaking within one to two hours of symptom onset [15]. Its sensitivity is limited, and normal values do not reliably exclude anaphylaxis. In this case, tryptase was within normal institutional range, but as significantly lower baseline tryptase was noted on outpatient follow-up, the perioperative tryptase values support anaphylactic reaction. Measurement of a baseline serum tryptase level at outpatient follow-up may aid in interpretation. 

The mechanism responsible for medication-related anaphylaxis may be IgE-linked or related to mast cell activation. Though there is variation based on global location, about half of allergic reactions in the pediatric operative environment are due to antibiotics, one-third are due to neuromuscular blocking medications, and about 10% are due to chlorhexidine [16]. Of note, prior exposure to a neuromuscular blocking agent or other medication is not necessary for a patient to develop anaphylactoid reactions, as there are other epitopes in the environment that can encourage formation of a response to medications [17].

There are significant differences in reported rates of allergic reactions under anesthesia depending on the country and study, but rates range from 1:1700 to 1:37,000. In a report on the Wake-Up Safe database by Wakimoto et al. regarding pediatric anaphylaxis during anesthesia, there was an 11% incidence of CPR and a 1.6% fatality rate [18]. This review also noted that the most common culprits were neuromuscular blocking medications and antibiotics, additionally noting opioids as a key causative agent. While most allergic reactions are noted in the operating room, it is possible for them to also manifest in the recovery room (especially after topical antibiotics or medications used at the end of the case). Almost all (97%) cases required escalation of care, and 91% were considered not preventable.

The mainstay of treatment for allergic reactions is epinephrine, and additional medications such as vasopressin and antihistamines may be used to support hemodynamics and reduce the allergic response, respectively. Administration of epinephrine in a timely fashion is ideal, as delay may contribute to worsening cardiopulmonary compromise.

## Conclusions

Anaphylaxis in the operative setting must be recognized and treated promptly. Epinephrine is the mainstay of treatment. The lack of pathognomonic symptoms such as hives and bronchospasm may make the diagnosis more challenging. Some hypotension may be encountered after routine administration of induction-dose anesthetics and may also reduce initial suspicion for anaphylaxis; in this case, initial hypotension was treated with phenylephrine, but the patient's condition did not improve until epinephrine treatment was initiated.

Additionally, p-PEA may initially be underrecognized in the presence of reassuring monitoring data, as was the case with this patient, whose end-tidal capnography and pulse oximetry data did not appear concerning. Patient skin mottling in the presence of hypotension not responsive to phenylephrine prompted consideration of anaphylaxis and a severe hypoperfusion state requiring epinephrine and CPR. Definitive treatment of the anaphylactoid reaction resolved the p-PEA, and the patient made a full recovery.
